# Association between serum free fatty acid levels and possible related factors in patients with type 2 diabetes mellitus and acute myocardial infarction

**DOI:** 10.1186/1471-2261-14-159

**Published:** 2014-11-14

**Authors:** Zhi-Hua Lv, Pei Ma, Wan Luo, Hui Xiong, Lu Han, Si-Wei Li, Xin Zhou, Jian-Cheng Tu

**Affiliations:** Department of Clinical Laboratory Medicine and Center for Gene Diagnosis, Zhongnan Hospital of Wuhan University, Wuhan, Hubei 430071 China

**Keywords:** Acute myocardial infarction (AMI), Type 2 diabetes mellitus (T2DM), Free fatty acid (FFA), High density lipoprotein cholesterol (HDL-c), Aspartate aminotransferase (AST)

## Abstract

**Background:**

Free fatty acids (FFAs) play importance roles in the development of diabetes and cardiovascular diseases. We measured serum FFA levels from type 2 diabetes mellitus (T2DM) and acute myocardial infarction (AMI) patients and assay the correlation between serum FFA levels and related factors. The present study was undertaken to investigate a possible relation between the changes in serum free fatty acid concentration with acute myocardial infarction and type 2 diabetes mellitus.

**Methods:**

The study population consisted of 540 healthy individuals and 103 patients with T2DM, 59 patients with AMI and 21 volunteers. Serum FFAs were measured with high pressure liquid chromatography. Blood urea nitrogen and uric acid were measured in clinical laboratory, as were glycemic, lipid and blood routine parameters. We selected 242 individuals with age over 60 years, 143 healthy individuals and 52 patients with T2DM, 47 patients with AMI were incorporated into three groups as control group, T2DM group and AMI group. Associations were analyzed with stepwise regression analysis with adjusted for age, sex, body mass index.

**Results:**

Serum FFA levels were significantly higher in the age over 60 years individuals compared to 20 ~ 50 years (logFFA μmmol/L:2.60 ± 0.16 vs. 2.73 ± 0.18, P < .001) in the healthy group. We found lower FFA levels in the AMI compared to the T2DM and control group (2.64 ± 0.22 vs. 2.72 ± 0.13&2.72 ± 0.16, respectively, P < .05&P < 0.01) in the age over 60, fasting blood glucose level higher in the AMI and T2DM (5.78 ± 1.32&7.75 ± 2.93 mmol/L vs. 4.90 ± 0.47 mmol/L, P < .01&P < .001) compared with the normal group, HDL level (1.01 ± 0.22&0.98 ± 0.18 mmol/L vs.1.30 ± 0.22 mmol/L, P < .001&P < .001). With stepwise regression analysis, the serum FFA levels was positively associated with the HDL in the control group (Y_logFFA_ = 2.32 + 0.33X_HDL_, R = 0.26, P < .01) and T2MD (Y_logFFA_ = 2.46 + 0.27X_HDL_, R = 0.36, P < .05), AST in AMI (Y_logFFA_ =2.24 + 0. 015X_AST_, R = 0.49, P < .01).

**Conclusions:**

Compared to control group, serum FFA levels were decreased only in AMI group, while HDL level was increased in both AMI and T2DM group. The serum FFA levels were positive association with the HDL level in both T2DM and control group, FFA levels were positive association with AST in AMI.

## Background

More than 347 million patients had diabetes around the world in 2008 [1], the World Health Organization projected that diabetes was likely to become the 7th leading cause of death in 2030 [2]. Diabetes increases the risk of cardiovascular disease and stroke, about 50% of people with diabetes die of cardiovascular disease [3]. Diabetes-associated atherosclerosis has been estimated to affect 5-8% of the general population and was by itself a major cause of death among diabetic patients with accounting for 50% of mortality [4]. Dyslipidemia contributes to diabetes-associated atherosclerosis and even to insulin resistance [5], and strong evidence supports the role of free fatty acids (FFAs) in promoting insulin resistance [6]. Insulin resistance increases atherogenesis and induces proinflammatory activities on vascular. Hyperglycemia caused feedback loop, increasing lipolysis and leading to a chronic exposure to the FFAs [6–8].

Cardiovascular diseases (CVDs) have emerged as the dominant contributor to total global, and the total number of CVDs deaths increased from 14.4 million in 1990 to 17 million in 2008. More than seven million patients are reportedly diagnosed with AMI each year, 90% of myocardial infarctions are attributable to modifiable risk factors as smoking, dyslipidemia, hypertension and diabetes [9]. Some studies have recently reported that STEMI patients with high glucose are related to adverse impact on survival, and the plasma FFAs concentration has been associated with lip toxicity, apoptosis, and risk of diabetes mellitus and coronary heart disease [10].

Circulating FFAs are albumin-bound lipid molecules principally derived from adipose tissue lipolysis [11]. The FFAs is a major fuel in mammals besides glucose. It is established that the excess supply of FFAs could suppress food intake and reduce hepatic glucose output. An overabundance of FFAs, due to exogenous lipid infusions or obesity, can lead to insulin resistance, vascular dysfunction, and myocardial dysfunction. Additional studies are needed to explore biological mechanisms that FFAs may influence the happen of AMI.

## Methods

### Subjects

This study was carried out at the Zhongnan Hospital of Wuhan University and Renmin Hospital of Wuhan University. The research protocol was approved by the Medical Ethics Committee of the Zhongnan Hospital of Wuhan University. All individuals provided informed consent with verbally or in writing. The normal group population was selected from those who underwent a comprehensive health examination at Zhongnan Hospital of Wuhan University, Renmin Hospital of Wuhan University. The study subjects included 103 T2DM and 59 AMI patients who had been hospitalized at two departments: Department of endocrinology and cardiovascular medicine in Zhongnan Hospital of Wuhan University. The 21 volunteers were graduate students from the Center for Gene Diagnosis of Zhongnan Hospital of Wuhan University. The health examination individuals were selected for participation based on the following criteria: liver, kidney function and blood test were in the normal range for the most parameters, and without other systemic disease history.

Type 2 diabetes was defined according to the American Diabetes Association (ADA) [12]. Any one of the following criteria meets the diagnosis for T2DM (In the absence of unequivocal hyperglycemia, criteria 1–3 should be confirmed by repeat testing): (1) HbA_1c_ ≥6.5%. (2) FPG ≥7.0 mmol/l. (3) Two-hour plasma glucose ≥11.1 mmol/l during an OGTT. (4) In a patient with classic symptoms of hyperglycemia or hyperglycemic crisis, a random plasma glucose ≥11.1 mmol/l. Patients were excluded for the following reasons: (1) Patients who were diagnosed with type 1 diabetes mellitus (2) Patients who were complicated with cardiovascular disease, viral hepatitis, tumor, autoimmune disease, serious liver or kidney dysfunction (3) patients who use lipid-lowering drugs (e.g. Lipitor and Zocor, etc, which are in a class of drugs known as statins).

AMI was defined according ESC/ACCF/AHA/WHF [13, 14]. Detection of a rise of cardiac troponin (cTn) with at least one above the 99th percentile upper reference limit (cTnT ≥0.01 ng/L) and with at least one of the following: (1) Symptoms of ischaemia: chest pain lasting 30min or longer. (2) New or presumed new significant ST-segment-T wave (ST-T) changes or new left bundle branch block (LBBB). (3) Development of pathological Q waves in the ECG. (4) Imaging evidence of new loss of viable myocardium or new regional wall motion abnormality. (5) Identification of an intracoronary thrombus by angiography or autopsy. Patients were excluded for the following reasons: (1) Previous myocardial infarction. (2) Patients who were complicated with diabetes mellitus, viral hepatitis, tumor, autoimmune disease, serious liver or kidney dysfunction. (3) Patients who used lipid-lowering drugs (e.g. Lipitor and Zocor, etc. which are in a class of drugs known as statins).

### Study population

Height (cm) and weight (kg) were measured in the standing position when health examination or admission. Venous blood samples were collected after overnight fasting for T2DM patients, healthy examination individuals and volunteers. Venous blood samples were collected for AMI patients within 4 hours of admission. Serum samples were prepared by centrifugation at 2000 rpm for 10 min at 4°C. Blood routine parameters were measured using the automatic hematology analyzer (Beckman-CoulterLH750, USA). The inter-assay coefficients of variation (*CV*%) amounts to 3.6% and 2.9% in the higher normal and lower normal range respectively. Fasting serum glucose (GLU), total cholesterol (TC), triglyceride (TG) and high-density lipoprotein cholesterol (HDL-c), low-density lipoprotein cholesterol (LDL-c), Serum aminotransferase (ALT) and aspartate aminotransferase (AST), serum urea nitrogen(BUN), uric acid concentrations were measured using the automated chemistry analyzer (Olympus AU5400 biochemistry analyzer, Japan). The inter-assay coefficients of variation (*CV*%) amounts to 3.9% and 2.7% in the higher normal and lower normal range respectively. Serum samples for FFAs assays were stored at -80°C until analysis. Serum FFA levels were measured with high pressure liquid chromatography (HPLC) within one hour after rehydration.

### Statistical analysis

SPSS 13.0 software was used for statistical analysis. Categorical variables were expressed as proportions and compared between groups using the X^2^ test. Continuous data are expressed as mean ± SD for normally distributed variables or median (inter quartile range) for others. The paired t-test was used to compare continuous variables. Unavailable relationships were calculated with Pearson correlation coefficients. Comparison of the groups by ANOVA was followed by SNK-q test to determine differences between individual groups. Multivariable stepwise regression analyses were performed to determine the independent correlation factors to FFA. Because of skewed distribution, the values of FFA were transformed in logarithmically for analysis. The level of significance was set at P < .05.

## Results

### The stability of FFAs

**Figure 1:**
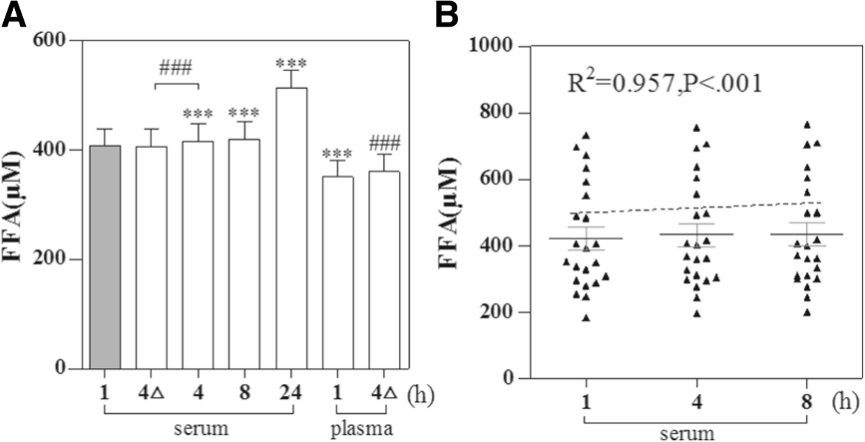
**The change of FFA levels with different storing temperature and time.** The histogram showed FFA levels **(A)** 4△represents stored at 4°C 4 h, others stored at room temperature .Scatter plots show relationships of storing time (h) and serum FFA **(B)**. ***P < .001, paired t-test: compared to room temperature 1 h. ^###^P < .001, paired t-test: compared to 4°C 4 h. The dotted line was a virtual-line.

A total of 21 volunteer subjects were enrolled in this study. Mean age was 26.7 ± 2.3 years, and 33% were men. The serum sample was stored at room temperature (1 h, 4 h, 8 h, 24 h) and at 4°C 4 h, and plasma sample at room temperature 1 h and at 4°C 4 h. There was no significant difference in serum FFA levels between the room temperature 1 h and 4°C 4 h (407.3 ± 148.4 mmol/L vs. 406.2 ± 148.1 mmol/L, P = 0.19). The FFA levels began to increase in those groups under room temperature (4 h: 416.9 ± 149.2 mmol/L; 8 h: 419.5 ± 149.2 mmol/L; 24 h: 513.8 ± 151.2 mmol/L; P < .001) compared to room temperature 1 h (407.3 ± 148.4 mmol/L) (Figure 1A). Plasma FFA levels were significant lower compared to the serum levels (room temperature 1 h: 351.3 ± 137.9 mmol/L vs. 407.3 ± 148.4 mmol/L; P < .001).The scatter plot also showed an elevated of FFA levels with the storing time lasted (R^2^ = 0.957, P < .001, Figure 1B).

### The FFA levels in different age and gender

**Figure 2:**
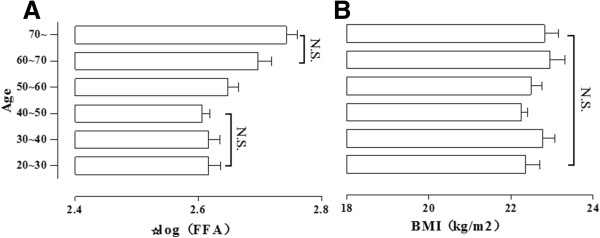
**The change of FFA levels in different age.** FFA levels **(A)** and BMI **(B)** in different group. ANOVA was followed by SNK-q test to determine differences between individual groups; Data are mean ± SD (N.S.: no statistically significant.

We divided 540 healthy individuals into six sub-groups (Figure 2). There was a significant difference among the six sub-groups after adjusted for sex and BMI, the FFA levels had a trend of increasing with the age, but not enough to achieved statistically significant among the age among 20 ~ 50 years and 60 ~ years. We found no difference between sexes (Table 1).

**Table 1:** **The FFA levels between sexes**

Sex	Age (y)					
	n	20 ~ 50	n	50 ~ 60	n	60~
Male	145	2.60 ± 0.16	56	2.65 ± 0.18	75	2.72 ± 0.18
Female	153	2.61 ± 0.16	55	2.65 ± 0.19	56	2.73 ± 0.17

Independent-Samples T Test.

### Baseline characteristics

Baseline characteristics were shown in Table 2. A total of 242 eligible consecutive individuals were included which age was over 60 years between June 2013 to October 2013. There were 143, 52 and 47 subjects in the control group, T2DM and AMI, respectively. All data were in a normal distribution exclude ALT, AST and FFAs.

**Table 2:** **Baseline characteristics**

	Control group	T2DM	AMI
**Age** (y)	71.0 ± 6.6	70.9 ± 7.3	71.3 ± 7.0
**Males,** n (%)	81 (56.6%)	24 (46.2%)	24 (51.1%)
**BMI** (kg/m2)	22.5 ± 2.6	23.5 ± 2.3**	22.3 ± 3.0
**Hypertension,** n (%)	15 (10.6%)	9 (17.0%)	22 (46.8%)
**ALT** (U/L), M (P25,P75)	20 (17,25)	23 (19,29)**	23 (19,31)^##^
**AST** (U/L), M (P25,P75)	25 (22,28)	23 (18,29) **	25 (21,30)
**Total Cholesterol** (mmol/L)	4.31 ± 0.59	4.59 ± 1.03*	4.40 ± 0.90
**Triglycerides** (mmol/L)	1.12 ± 0.33	2.02 ± 1.29***	1.63 ± 0.88^###^
**HDL-cholesterol** (mmol/L)	1.30 ± 0.22	0.96 ± 0.18***	1.00 ± 0.22^###^
**LDL-cholesterol** (mmol/L)	2.35 ± 0.46	2.37 ± 0.77	2.25 ± 0.75
**Glucose** (mmol/l)	4.90 ± 0.49	7.75 ± 2.95***	5.78 ± 1.32^###^
**BUN** (mmol/L)	5.50 ± 1.28	5.78 ± 3.13	6.22 ± 2.82^#^
**Uric acid** (mmol/L)	281.3 ± 68.2	316.2 ± 96.9*	366.3 ± 171.7^###^
**HCT**	41.6 ± 3.6	38.5 ± 5.2***	38.8 ± 4.9^###^
**WBC** (10*9/L)	6.15 ± 1.60	7.30 ± 3.78**	7.12 ± 2.41^#^
**Platelet** (10*9/L)	178.7 ± 49.0	180.8 ± 63.0	182.0 ± 74.0
**Total Protein** (g/L)	71.1 ± 6.0	67.4 ± 6.0*	66.5 ± 6.6^##^
**FFAs** (mmol/L)	551.6 (403.0,679.7)	536.5 (412.6,666.3)***	441.0 (298.5,625.0)

Data are presented as mean ± SD, in a normal distribution of data, or as median (inter quartile range), in a skewed distribution of data.

*P < .05, **P < .01, ***P < .001for the T2DM group vs. the control group.

^#^P <0.5, ^##^P < .01, ^###^P < .001 for the AMI group vs. the control group.

### Associations of FFAs with other conceivable factors

**Figure 3:**
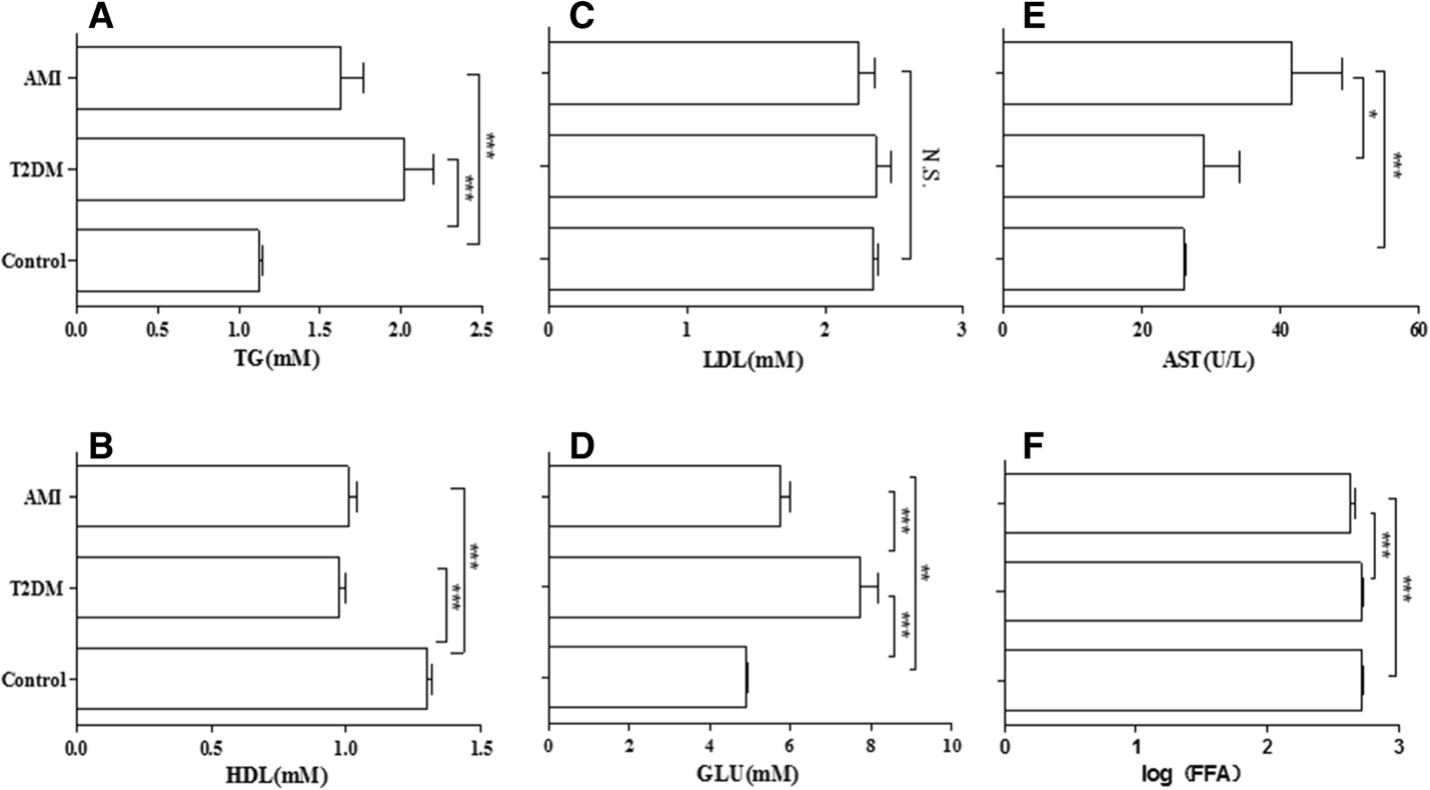
**The description of six mainly levels in T2DM, AMI and control group.** The histogram showed six mainly levels [TG **(A)**, HDL **(B)**, LDL **(C)**, GLU **(D)**, AST **(E)**, FFAs **(F)**] Data are mean ± SD. *P < .05, **P < .01, ***P < .001; Three groups: Control (n = 143), T2DM (n = 52), AMI (n = 47).

In this part, 242 subjects were chosen according to age greater than or equal to 60 (129/242 were men; 71.0 ± 6.8 years). There were 143, 52 and 47 subjects in the control group, T2DM and AMI, respectively. We compared the three groups with one-way ANOVA (Figure 3). Serum levels of the triglyceride (TG) and glucose (GLU) were significantly higher in individuals with T2DM and AMI compared to control, as TG (2.02 ± 1.29 mmol/L&1.63 ± 0.88 mmol/L vs. 1.12 ± 0.33 mmol/L, P < .001&P < .001) (Figure 3A) and glucose (7.75 ± 2.93 mmol/L&5.78 ± 1.32 mmol/L vs. 4.90 ± 0.49 mmol/L, P < .001&P < .01) (Figure 3D). The HDL levels were lower in T2DM (0.98 ± 0.18 mmol/L, P < .001) and AMI (1.01 ± 0.22 mmol/L, P < .001) compared to control group (1.30 ± 0.22 mmol/L) (Figure 3B). And serum FFAs and AST obtained significant difference in AMI compared to T2DM or control (P < .001) (Figure 3E and F).

The participants were divided the participants into four groups based on the age as shown in Table 3. Stepwise multivariable regression models were used to find the association of FFA with each of the other factors with adjusted sex and BMI. Those factors ALT, AST, TC, TG, HDL, LDL, BUN, TP and fasting glucose were incorporated into the regression models.

**Table 3:** **Stepwise multivariable regression model of the serum FFA levels**

	Males, n (%)	Age (y)	Log (FFA)	FFA	
				R	P-value
**Control** (20 ~ 50 years)	145 (48.7%)	39.3 ± 8.6	2.61 ± 0.16		
**HDL + TG**				0.28	P < .001
**Control** (≥60 years)	81 (56.6%)	71.0 ± 6.6	2.72 ± 0.16		
**HDL**				0.26	P = 0.002
**T2DM** (≥60 years)	24 (46.2%)	70.9 ± 7.3	2.72 ± 0.13		
**HDL**				0.36	P = 0.01
**AMI** (≥60 years)	24 (51.1%)	71.3 ± 7.0	2.64 ± 0.22		
**AST**				0.49	P = 0.005

For stepwise multivariable regression analysis, we selected the variables that were allowed to enter the model in advance. HDL and TG were associated with The FFAs in the control group (20 ~ 50 years) (R = 0.28, P < .001) and were allowed to enter the multivariable model, so was HDL was associated with FFAs in the control group (≥60 years) (R = 0.26, P < .01) and T2DM (≥60 years) (R = 0.36, P < .05), AST was associated with The FFAs in the AMI (≥60 years) (R = 0.487, P < .01).

## Discussion

The current study demonstrates that nearly two-thirds of patients with cardiovascular disease suffer from abnormal glucose metabolism [15, 16], and type 2 diabetes is associated with a twofold to fourfold increased risk of both coronary heart disease and stroke [17]. Cardiovascular disease was also the most common underlying cause of death in type 2 diabetes mellitus accounting for 52% [3].

In this study, the FFA levels had a trend of increasing with age, and no significant difference between sexes. Preliminary experiments show the FFA levels were instability in room temperature, and the difference between serum and plasma sample was also found. Also, the detection of FFAs is limited by the significant intra-individual range of variation, which can be strongly influenced by food intake and stress situations [18]. All individuals were included in the analysis of the age greater than or equal to 60. In stepwise multivariable regression, the FFAs were positive correlated with HDL in the health examination individuals or T2DM, however, no similar correlation in the AMI. And lower FFA was strongly associated with AST in the AMI.

FFAs serve as physiologically important energy substrates and their release from the adipose tissue by lipolysis is regulated according to the energy demands of the body. FFA elevations are both a risk factor of cardiovascular disease and are associated with metabolic, atherosclerosis, inflammation, insulin resistance and hyperlipidemia [19–23]. The 70% of energy was generated through β-oxidation of fatty acids and 30% through oxidation of ketone bodies and pyruvate in the cardiomyocytes [24, 25]. A raised concentration of FFAs with subsequent increased β-oxidation in the cardiomyocytes can be harmful for the heart in various ways. Furthermore, increased FFAs can lead to damage to the plasma membrane and disturbances of the ion channels of the cardiomyocytes [25]. The fatty acid levels has been described an increase in myocardial infarction [26], however, we found the free fatty acid levels has a slight decrease in the early period of acute myocardial infarction, it may play an important role in the early stage of a first-time AMI.

The ratio of low-density lipoprotein cholesterol and high-density lipoprotein cholesterol (LDL-c/HDL-c) is a reliable predictor of cardiovascular risk. Low HDL-C levels in patients with coronary artery disease are associated with a high risk for cardiovascular events [27]. LDL-c and non-HDL-c are likely to be the predictors of cardiovascular risk and as targets for treatment [28, 29].

The liver transaminases, especially aspartate transaminase (AST), were first found their use in myocardial injury and necrosis in 1954 [30]. A total of 1783 patients study: AST was elevated above the upper limit of normal in 85.6% in acute myocardial infarction, CK-MB was association with AST (r = 0.727), and independently associated with worse mortality and clinical outcomes [31]. FFAs may serve as physiologically important energy in AMI patients, and play as protective factors during the acute attack period of the AMI.

## Conclusions

In conclusion, we found lower serum FFA levels in AMI during the acute attack period compared to the other two groups, independent of age, sex, BMI, the serum HDL levels were lower in both AMI and T2DM compared to control group, and AST levels were higher in AMI compared to control group. With stepwise multivariable regression analysis: the HDL was associated with the FFAs in control group and T2DM patients, but the AST was associated with the FFAs in the AMI, the FFAs possibly play importance roles in the AMI.

## Abbreviations

FFA: Free fatty acid
T2DM: Type 2 diabetes mellitus
AMI: Acute myocardial infarction
BMI: Body mass index
HPLC: High pressure liquid chromatography
ALT: Serum aminotransferase
AST: Aspartate Aminotransferase
HDL-c: High-density lipoprotein cholesterol
LDL-C: Low-density lipoprotein cholesterol
TC: Total cholesterol
TG: Triglyceride
GLU: Fasting serum glucose
BUN: Serum urea nitrogen
WBC: White blood cell.
